# Quarterly Periscope, Extra

**Published:** 1823-03-01

**Authors:** 


### Quarterly Periscope

OF

### PRACTICAL MEDICINE;

BEING THE

### Spirit of the Public Journals,

FOREIGN AND DOMESTIC.

*Paucis libris immorari et innutriri oportet, si velis aliquid trahere, quod in animo fideliter hæreat.* SENECA.

Duo vitia vitanda sunt in cognitionis et scientiæ studio. ***** Alterum est vitium, quod quidam nimis magnam operam conferunt in res obscuras atque difficiles, easdemque non necessarias. CICERO.

### EXTRA LIMITES.*

### *On the Treatment of Fractures of the Lower Extremity.* By J. AMESBURY, of the Royal College of Surgeons, London.†

THE author states that it is not his intention to give a detailed account of the treatment of fractures of the leg and thigh at present, but merely to throw out a few observations, which he has found useful in his own practice.

He goes on to enumerate some of the principal plans of treatment that have hitherto been adopted by different practitioners; and conceives that the employment of any one of them is attended with disadvantages to the patient, which materially militate against its use; and then rapidly proceeds to the consideration of the causes of dis-

---

* The great number and length of our Analytical Reviews this quarter has left us no room for our usual Quarterly Periscope; nevertheless we have given an Extra-limites Periscope, or Analytical Review of an important paper on fractures, by Mr. Amesbury, at our own private expense. We have also to state, that the number of applications which we have had for the insertion of trifling, unimportant, or self-interested papers, in our Extra Limites, has determined us to fill up that department, in future, with *extra-analyses*, as on the present occasion—a circumstance that will save us much tedious private correspondence, for which we have not leisure, and render the Journal uniform and homogeneous.

A few of our correspondents, from whom we received some good papers, must take this as an apology for not complying with their wishes.

† See Quarterly Journal of Foreign Medicine, No. XV.

placement. In these, he thinks, the bones themselves in no way participate. Among these causes the action of the muscles is considered the principal. Every other he conceives to be secondary or accidental. The action which the muscles exert upon the broken bones is not looked upon as voluntary, but as altogether depending upon their tonic power. This kind of contraction is much increased by every thing that irritates them ; and as the fractured ends of the bones, when once displaced, oppose their ragged edges to the inflamed and tender bellies of the muscles, they must be regarded as a powerful mechanical stimulus, which occasions the muscles to contract more forcibly, and increase the displacement and all its attendant evils. From this it would appear that one grand principle in the treatment of fractures is, to place the bones as early as possible in a natural position, and a second is, to keep them from moving and injuring the soft parts.

With due deference to his predecessors, the author conceives every man has a right to think for himself; and from his reasoning, it might be inferred, that no one, however celebrated he may be, is to be considered culpable for not giving the best plans of treatment for the disease or accident upon which he writes. The surgeon does his duty as an author, both to his brethren and the public, if he lays before them the best plans he is acquainted with, or such as he conscientiously believes to be correct. If any one points out a mode of treatment, which, on fair examination, shall be found preferable to those previously adopted, he confers an obligation on every member of society. He advances the profession one step nearer to perfection, and the honor due to him is not to be taken away by any thing his successors may bring forward. A complex being is not to be viewed at a single glance. Many parts of our profession require for their development the talents of many ages.

Wishing to add something to the general stock, the author proceeds "to offer a few observations, which may tend to throw some light on the mechanical treatment of injuries, of so much interest to the patient as well as to the surgeon."

Speaking of the straight position in fractures of the thigh, he says: —" To judge correctly of the proper position of the limb, in a case of fracture, we must take into consideration the natural form of the fractured parts ; and, also, those powers which tend to displace the bone when broken. What then, for instance, is the natural form of the femur? not straight, as the practice of placing the limb in the straight position would indicate, but it forms a segment of a large circle, whose convexity is placed before, and concavity behind. The points of support, therefore, when this bone is placed upon a plane, with its concavity towards it, are at the ends ; and the effect of fracture through its middle, would be to divide the segment of a circle, which the thigh-bone forms, into two smaller segments; and the middle of the bone being unsupported would reach the plane; and the lower edges of the fractured ends will be seen exposed, and ready to prick and lacerate the surrounding textures."

This is, we think, the fairest view that can be given of this practice. It supposes the bone to be uncovered by the soft parts, and merely broken through the middle as it lies upon a plane; and it shews that, if the muscles were perfectly quiescent in a case of fractured thigh, the practice of placing the limb in the straight position, is not borne out by the anatomy of the parts, when the fracture is through any part that might become thus displaced by the simple want of continuity, independent of the action of the muscles; and it is an argument that goes to prove, that the middle of the bone ought to be supported under all positions and motions of the limb.

We now pass on to the consideration of displacement, in fractures of the thigh, as it is observed to be influenced by the action of the muscles, when the limb is placed in the straight position; and this is greatly modified by the degree of laceration of the soft parts "at the site of fracture; the situation of the fracture; its direction, &c."

"When the thigh is amputated high up, the flexors often act so powerfully as to place the stump, for a time, nearly at right angles with the pelvis, hence it must be evident, that when there is a solution of continuity in the bone, the muscles which tend most to displace the upper fragment, are the iliacus internus and psoas magnus; and the effect of their contraction is more or less powerful in raising the upper part, in proportion to the distance of the fracture from the point of their insertion; or the length of the lever by which they are resisted. But what muscles act most upon the lower portion, so as to produce displacement in the transverse direction? It will be recollected, that the fixed points for the action of the gastrocnemius and popliteus are, in the natural state, principally the condyles of the femur; and that their contraction assists in the extension of the foot and flexion of the leg. But fixed points, in anatomical language, are considered as those which oppose the greatest resistance to the action of muscles, hence it will appear that those points, which in the natural state are the most fixed, may after the accident become the most moveable. If a muscle were attached to two bodies, which oppose an equal degree of resistance to a contraction of its fibres, these two bodies must move with an equal degree of velocity towards its centre; but if one of these bodies be more fixed, either by weight or length of lever, than the other, the lighter body must move towards the heavier, or more resisting, in whatever manner the resistance may be produced. From this it will appear, that when a fractured thigh is placed in the extended position upon the heel, the fixed points for the action of the gastrocnemius and popliteus are, pro tempore, no longer in the condyles, but at the points of their insertion; therefore, the contraction of these muscles tends to carry the lower fragment in a direction directly opposite to that of the upper; and this with a degree of force proportionate to the distance of the fracture from the lower end of the bone. If the solution of continuity be across the upper third, the transverse displacement, as far as the muscles are concerned, is principally produced by the action of the iliacus internus and psoas magnus; and if near or through the condyles, by that of the gastrocnemius and popliteus:—the former muscles move the part to which they are attached upward and forward; and the latter, downward and backward; thus, the upper portion is bent upon the pelvis; and the lower is bent upon the leg. The straight position puts these muscles intò a state of tension, and consequently increases their power over the unresisting fragments. The transverse displacement being once produced, the extensors and long flexors of the leg are goaded into action; and, assisted by the triceps adductor femoris and, if the fracture is high up, by the pectinæus, they contract forcibly and draw up the lower portion, which glides beneath the upper —sometimes the distance of several inches, and, at last, comes in contact with it at a considerable angle. From these causes arise pain, spasm, inflammation, abscess, deformity and lameness."

"Other muscles tend to produce displacement, according to the situation of the fracture, but these I need not mention, as my object was merely to shew those *muscles which principally* produce displacement in the *transverse direction*, a displacement which must necessarily precede shortening of the limb."

We are not told in what way the action of the muscles affect the fracture, when the soft parts are much torn; or when the bone is broken in any particular direction. The cause of this omission may be looked for in the onset, where the author states it is not his intention to enter into detail at present; we must, therefore, wait the complete development of his notions upon this subject, before we venture to make our own observations upon them.

"Mr. Pott had the honor of pointing out the advantages we gain by bending the limb, and consequently relaxing the muscles, which tend most to disturb the fracture; and to forward his views upon this subject, he laid the limb upon the side in the half bent position. In this posture, the limb may be placed at any angle the case may require; and forms, from the trochanter to the foot, a line straight enough to lie sufficiently smooth upon a plane; provided the trunk, also, is placed upon the side; and, if the patient could maintain this position during the period of cure, the limb may be straight and perfect; but he soon becomes tired and turns upon his back. By this rotatory motion, the trunk carries with it the upper fragment of the fracture, while the lower fragment and the leg continue as they were placed; and hence arises eversion of the foot:—a species of deformity which, in this kind of treatment, is with difficulty avoided, and is every day occurring."

From what we have here extracted we perceive, that the author approves of Mr. Potts' plan of relaxing the muscles, though he does not agree with him in the practice of placing the limb upon the side, according to the manner Mr. Pott advised. But it does not appear that the author wishes to argue against the practice of placing the limb either on the heel or on the side, but against confining it to either of those positions; for he says, (p. 442)

"But whoever attends strictly to the treatment of these injuries will find, that any one position long-continued is extremely irksome to the patient, and in the young and irritable can scarcely be maintained." And again, (in p. 443,) "I would contend not however for the practice of placing the limb upon the heel or upon the side, but for that practice which relaxes the muscles, and allows the patient himself to place it, according to his feelings, upon one or the other; and to alter it at pleasure, without any danger of disturbing the coaptation of the fractured parts."

He believes that the same objections arise in the treatment of fractures of the leg when placed upon the side, as in fractures of the thigh, though not so forcibly.

"When (p. 442) both bones (of the leg) are fractured and the limb is placed upon the heel, we daily see great difficulty experienced in keeping the fragments in apt and proper contact. The pads and pillows made use of cannot be with sufficient nicety adjusted to maintain the limb in that position, which a favourable result requires. The surgeon, therefore, at every visit, finds something to attend to;—the heel is raised too high, and the bones, consequently, bowed backwards; or it is sunk too low, and the fracture yields in an opposite direction. He is frequently obliged to replace his pads, pillows, brickbats, and other contrivances, made use of to supply the deficiency of his splints; and as frequently produces more or less motion between the fractured ends. This motion much retards the union, and is one of those causes which prevent it altogether."

Upon the cause of eversion he says—

"That the motion of the body in the direction I have mentioned is the cause of the eversion of the foot, will, I think, readily appear, by placing a person on his back, upon a plane, with the leg semi-flexed and laid upon the side. Unless the limb be very much bent, the thigh and upper part of the leg cannot come in contact with the plane upon which the person lies. The heel acting as a lever, keeps the foot everted, and thus, the foot and lower part of the leg, forming points of resistance to the action of the adductors of the thigh, maintain the limb in a twisted position. This position soon becomes painful if the limb is sound; but if a fracture exists, the patient is quickly relieved from the pain thus occasioned, by the rotatory motion which takes place between the fractured ends."

We pass from this hasty view relative to the positions of the limb, to the consideration of the mechanical powers hitherto made use of to keep the bones quiet and in apt and proper contact.

"Having cursorily pointed out some of the difficulties we have to encounter in the treatment of these accidents, let us now inquire if there be any means by which we can avoid them. But before we enter upon this subject, let us stay for a moment and consider wherein the means in common use fail to effect the desired end. The mechanical powers now commonly adopted, to fix the fractured parts, are either the short or long splints, the fracture-box, or doubly-inclined plane."

"In order to shew how far the short splints produce the effect for which they were intended, I made the following experiment:—I divided a stick, half an inch in diameter and two feet long, into two equal parts, and one of these I again divided into two parts of equal length. The two shorter pieces I intended to represent the two fragments of a fractured thigh; and the longer, the leg in a natural state. I then connected, by means of a hinge-joint, one end of the longer piece with one end of one of the shorter; and, having brought one end of each of the shorter pieces into contact, as they lay in a straight line, I surrounded the shorter pieces, their whole length, with muscular fibre, one inch and a half in diameter, placed in the longitudinal direction, and secured with small twine. Over the muscular fibre, I laid slips of deal, in the usual way of putting up a fractured thigh with short splints, and confined them with tapes moderately tight. Having laid the whole upon a plain surface, I rotated the longer piece, uncovered by muscular fibre, and found, as I expected, that the motion was lost between the shorter pieces; and that no motion was given to the upper of these, except what was communicated to it through the medium of the muscular fibre; and that the centre of every kind of motion, which did not loose itself in the hinge-joint, was placed between the approximated ends of the shorter pieces. Hence it appears, that when the thigh is broken and put up with short splints—by which I mean splints that extend from the pelvis to the knee—motion given to either end of the limb is lost in the site of fracture."

From this experiment, the author is induced to believe, that the short splints would not confine the fractured parts, however closely they may be applied; and, he thinks, if they are placed very tight upon the limb, they may force a portion of the surrounding textures between the broken ends of the bone; he, therefore, concludes, that these splints "fail to accomplish the purposes for which they were constructed:—they fail to maintain the proper coaptation of the fractured parts, and to prevent any motion between them."

"What I have here stated with regard to the short splints for fractures of the thigh, will also apply, in some measure, to those in common use for fractures of the leg. They do not provide against motion, which impetus given to the limb produces in the fracture; there is nothing in their construction to support the heel and sole of the foot; parts which, in the treatment of these accidents, deserve our serious attention; nor, unless they are kept very tight upon the limb, is the lever above the knee sufficiently long to guard against eversion.

"They are simple, however, and easy of application; but, when we have an important object to accomplish; when the limbs or the lives of our fellow creatures are in danger, utility and efficacy should be borne in mind. Is it no small matter to suffer the pain and inconvenience arising from these injuries, and afterwards to be deformed and lamed for the remainder of our existence? It has been said that we should bring down our mechanical contrivances to the comprehension of the dullest capacity. This is a good rule and should be, as far as possible, adhered to; for simplicity, combined with utility, is excellency in these things. But if I were called upon to define what I mean by the term simplicity, as applied to a piece of machinery, I should say, it signifies that which answers the same intentions by the fewest means; or that which has in it all that is useful, and nothing superfluous; and I cannot help thinking that surgery stands in need of this kind of simplicity in the construction of its instruments.

" If we consider the motions of the limb in the natural state, we shall find, that the leg is passive to the motions of the foot; the thigh to the motions of the leg; and the pelvis to the motions of the thigh; hence we should infer, that if the foot and thigh were fixed by a continuity of splint, the leg must of necessity become fixed also; and if the foot, leg, and pelvis could be fixed, so that no motion could take place in the one without passing over to the other, the thigh also would become a fixture.

" To illustrate this point, and to see what we gain by fixing the whole limb in the treatment of fractures, I covered, not only the shorter pieces of the stick abovementioned, but also the longer with muscular fibre, while they remained in the same relative position as described in the last experiment; and having fixed a cross bar to the lower end of the longer portion, I applied slips of splint two feet long—the length of the three pieces—upon the muscular fibre, and confined them to this and to the cross-bar with small twine. The whole was now placed upon a plane, and rotated and moved in different directions, by applying the hand to the cross-bar; and it was found that any motion given to the cross-bar, which was intended to represent a foot-board, was not felt between the approximated ends of the shorter pieces, as in the first experiment, but was propagated over them, and lost at the upper end of the splints—a situation intended to answer to the hip-joint in a case of fractured thigh.

" This experimeut, rough as it is, is sufficient to show that when the thigh, leg, and foot are fixed, by any inelastic body, continued from the one to the other, motion between the fractured ends of the thigh bone, from an impetus given to the limb below the site of fracture, is effectually prevented.

" It will be seen, from what was stated in the first experiment, that the advantages here gained arose from fixing the splints to the cross-bar; hence it must be evident, that any motion given to the upper of the shorter pieces, which was in no way connected to the splints, by applying the fingers to its projecting end, would still have had its centre between the lower end of this piece, and that with which it came in contact. This is precisely what would occur in a fractured thigh, put up as the last experiment would indicate, if the pelvis could not move upon the femur; and if it were unconnected to the splints, which fix the knee and ankle. But it will be recollected that the pelvis has free motion upon the thigh in every direction, therefore it is not necessary that the upper fragment of the fracture should move, if the pelvis moves.

" Supposing the lower part of the limb not under the command of the patient, those movements of the pelvis which carry it out of a line with the thigh bone, must necessarily affect the fracture; but if the whole limb is fixed to the hip-joint, and the fracture is not so high up as to prevent the splints from holding the upper fragment firmly, the patient is able to direct its motions upon a plane whether the limb be bent or straight, and therefore can make it follow the movements of the pelvis, and consequently has it in his power to avoid the displacement which might otherwise ensue.

"But it will be seen that if the pelvis could be fixed upon the thigh, by a continuity of the same means that fix the foot and leg, motion given to either end of the limb would be still less likely to affect the fractured parts. Supposing the limb with the thigh bone fractured put up as here suggested, motion given to the foot would not have its centre in the hip-joint, but would pass over it to the pelvis; and if given to the pelvis, it would not be felt in the site of fracture, but would be propagated along the splints to the foot, which however would not feel it, but in common with the leg and thigh; and as far as it regards the motions given to the limb from external causes, this would be the case whether there exists, between the pelvis and the foot, one fracture or a dozen. From this it would appear that any plan which would enable us to fix the pelvis upon the thigh, by the same means that fix the foot and leg, is in some respect desirable."

If we conduct our treatment of fractures upon the principles which the author has endeavoured to establish, he concludes, that we shall find no difficulty in the treatment of fractures of the leg; and that our treatment of fractures of the thigh will become comparatively easy.

In alluding to M. Desault and Mr. Pott's practice, the author conceives, that M. Desault employed the long splint, in fractures of the thigh, principally with a view to keep up extension in the straight position, and at a distance from the fractured bone; and that Mr. Pott placed the limb upon the side, in order to relax the muscles which tend most to displace the fracture; and, in this way, to prevent the necessity of applying an extending force.

From the inconveniences attending the straight position, and the bent in most fractures of the lower limb, as pointed out by the author, when treated according to the common plans, we should infer that neither Desault nor Pott would have been inclined to follow the particular lines of practice, which they adopted, if they had been in possession of more effectual means to fix the fractured parts.

Speaking of the common modes of treatment, the author believes that the use of the fracture box and doubly-inclined plane, is attended with fewer disadvantages than any other means he has hitherto seen applied for fractures of the thigh.

"Supposing the limb (448) to be placed in the erect position, the fractured ends of the bone would be drawn in opposite directions; the upper forward, by the action of the flexors of the thigh; the lower backward, by the action of the gastrocnemius and popliteus; therefore, by placing the limb upon a doubly-inclined plane, we gain three points of evident advantage:—we weaken and resist the action of those muscles which tend to displace the lower fragment; and, by bringing up the limb upon the pelvis, we relax, and consequently weaken the action of the iliacus internus and psoas magnus; and place the patient in a position most easily maintained.

"But there are some objections to the use of these instruments, which must not be passed over in silence.

"The fracture-box and doubly-inclined plane are mounted upon wide frames, which prevent them from sinking into the bed upon which the patient lies. It is not so however with the nates. These and the trunk by their gravity cause the bed to yield, and in proportion as this gives way, if the instrument is placed so as to support the back of the thigh at all, the upper part of the limb is forced against its corresponding end; and thus the instrument tends to assist the iliacus internus and psoas magnus in bending the upper portion upon the pelvis, while the lower portion and the leg continue as they were placed. The pressure thus produced frequently occasions pain, tumefaction, and sometimes abscess, at the upper and back part of the thigh; and if the fracture is high up, the action of the iliacus internus and psoas magnus, assisted by the pressure of the instrument, is too often followed by great and permanent shortening of the limb, great deformity, and lameness. Again, even if no other mischief should arise when the patient uses the bed pan, the upper fragment, following the direction of the pelvis, is raised from the instrument, and motion is produced in the situation of the fracture. Are not these indications, which point out most clearly that any machine made use of for fractures of the leg and thigh, should be entirely passive to the motions of the limb?"

Upon the subject of extension in fractures of the thigh, we have the following observations:—

"It has been said that it is sometimes necessary to apply extension notwithstanding the limb is placed in the bent position. When this is required, I am disposed to believe, that the extending means should be made to act upon the lower end of the bone, or as near to it as possible; not however by applying a ligature round the limb, but by some means which would bring down the lower fragment, by acting upon a large portion of the thigh or back of the leg.

"To this plan I have not yet been able to discover any particular objection; and, therefore, think it preferable to extension in the straight position, to which the following may be started:—

"First, its effects upon the natural curvature of the thigh bone, which ought to be maintained. Second, it increases the power of the muscles which tend to produce displacement in the transverse direction. Third, it acts upon two sets of ligaments before the fracture is effected. Fourth, the natural figure of the limb forming curved lines on the inner and outer sides, is unfavourable to the practice."

These observations are so general, that it is impossible to say whether the author advises the extending means to be applied in all cases of fracture of the femur upon the thigh itself, and upper part of the back of the leg; but we should think not, as we have seen him place a fractured neck of the thigh bone in the straight position; and this being the case, it appears, from what we know of his plans of treatment, that the extending means must be made to act first upon the foot in fractures through the cervix.

After adverting to the advantages which accrue to the patient from raising the limb upon an inclined plane, when the inflammatory action is considerable, the author goes on to enumerate the following indications, which, from his own observations and experiments, he thinks should be attended to in the construction of any machine for fractures of the lower extremity.

" The instrument should—1st. Fix the whole limb so as to admit of no motion whose centre is not in the hip-joint, or between the pelvis and the back. 2d. It should maintain the fractured ends in a natural position, and in perfect coaptation. 3d. It should lie upon the limb with ease to the patient. 4th. It should enable the surgeon to place the limb at any angle the case may require. 5th. It should allow of extension and counter-extension when the limb is fixed in the bent position. 6th. It should be entirely passive to the motions of the limb, and should allow the patient to place it in any position most congenial to his feelings, either on the heel or on the side, and to alter this position at pleasure. 7. It should enable the attendants to move the patient from place to place, without any danger of displacing the fractured ends. 8th. It should allow of being adapted to limbs of different lengths and different sizes.. 9th. It should be applicable to fractures in any part of the limb, and of all kinds; whether simple, comminuted, or compound. 10th. It should be simple and easy of application. 11th. With all these advantages it should ensure to the patient a speedy recovery and a straight and perfect limb."

Mr. Amesbury has contrived a machine to answer these purposes, his own description of which we subjoin; and also his reasons for introducing into its composition each particular part.

" To fix the limb and allow it to be bent and fixed at different angles, a piece of beech slightly hollowed out, to receive the back of the thigh, thrown a little out of a straight line, to answer to the natural curvature of the thigh bone, and gradually narrowed towards the lower end, was connected by means of a hinge-joint, to another piece of beech, slightly excavated to receive the calf of the leg, and gradually narrowed towards the lower extremity, to answer to the natural form of the limb. From the situation for the calf to the inferior end this piece was made a little concave, simply to prevent the pad, upon which the limb is placed, from shifting its situation. Both of these pieces were cut out of one inch and quarter board; and that for the thigh was left one inch thick for two inches from its lower end, for the purpose of receiving, on each side, a brass-eyed screw; and that for the leg was left one inch thick, and the same width for six inches from its lower end. A bit of beech, a little longer and wider than the foot, was cut out at one end, to receive the leg-piece at right angles. This was intended for a foot-board. It was one inch thick and one broad, where it lay in contact with the sides of the leg-board; and when they were placed at right angles, the receiving end of the foot-board, and the received part of the leg-piece, were bored transversely, so that they may be fastened together by means of a bit of wire. That part of the foot-board which lay upon the concave part of the beech intended for the leg, was a little rounded off at the edge nearest the lower end of the leg-piece, so as to give it a hinge-like motion in a direction from right angles over the lower end of the leg-piece.

"To fix the pieces of beech intended to support the leg and thigh, and to allow of their being placed and fixed at different angles, I had a thin rod of steel, connected at one end, by means of a hinge-joint, to a short pillar of brass, which was fixed by screws to the middle of the back part of the leg portion. To the other end of this rod was fixed, by means of a hinge-joint, a brass foot, about an inch and half long, and one-fourth of an inch thick, with a hole in its centre, a little narrower in the transverve than in the longitudinal direction; and another hole in its side, which traversed the one in the centre transversely. To the side of the brass foot was fixed a bit of brass with a spring, in the form of a flute key, and at that end of the key which answers to the part that stops the hole in a flute, was fixed a steel pin, which was made to pass into the hole in the side of the brass foot, and cross the hole in its centre. A narrow piece of brass, with six stays projecting from its surface, was fixed to the back of the thigh portion. Each of these stays was of the form and size of the hole in the centre of the brass foot, connected to the steel rod, and each had a hole in it transversely to the plate, upon which they were arranged, large enough to admit the pin made to traverse the hole in the side of the brass foot. They were placed at a distance sufficient to allow the thigh piece to be fixed to that intended for the leg, at any degree of flexion, from straight to right angles.

"It will be seen that, by pressing the key, the pin attached to it, as above described, would be drawn out of the hole in the centre of the brass foot-piece, which could then be made to receive any one of the stays placed on the back of the thigh-piece; and once received, we have only to take the finger from the key, and the steel pin is forced, by the reaction of the spring, through the hole in the centre of the stay, and, consequently, the two pieces of beech may be fixed at any angle the case may indicate.

"To prevent the effects of moisture upon the steel rod, it is adviseable to have it coated with brass or tin, which may be easily done; but this is not required except for hospital practice.

"But it is proper that the instrument should be applicable to limbs of different lengths. The thigh and leg pieces must allow of being shortened more or less according to the length of the limb. To answer this purpose, as far as the leg piece is concerned, a number of holes of one size should be bored in a line transversely to this piece at its lower end; and then we have only to draw out the wire which fixes the foot-board to that intended for the leg, slide up the former upon the latter, and fix them where we please.

"The lengh of the thigh portion, however, is not so easily altered; but this may be done by a thin plate of brass the width of the thigh-piece, and five or six inches long, according to the size of the instrument. When hammered out to lie upon the hollow part of the thigh-piece, and turned off at its upper end, this plate had attached to it two thin plates or bars of steel of its own length, and about half an inch wide. As much of one end of each of the steel plates as was equal to the wood of the thigh-piece, was bent at right angles. They were then placed upon the back of the brass plate in the longitudinal direction, and the bent extrémities united to it, just below its upper end; and within half an inch of its sides. Having now placed the brass plate upon the thigh-piece, and the steel plates or bars behind it, I had nothing to do but unite the plate to the bars of steel through two long mortises in the wood, in such a manner as would enable me to slide the brass plate up and down, or fix it upon the wood at pleasure. To fix the brass plate and to allow it to slide, two small screws, with square heads, were passed through two holes at the lower end of the brass plate, opposite the steel plates or bars. These screws were then passed through the mortises in the wood; and through two holes of their own size made in the steel plates; where they were fixed by female thumb screws. By means of the female thumb-screws the brass plate could be fixed or made to slide upon the wood, so as to enable us to lengthen or shorten the instrument, according to the length of the femur; but this plate is not required except for fractures of the thigh and fractures, &c. of the leg, attended with high action.

"But legs are of different thicknesses as well as of different lengths; one man may have a larger calf or a longer heel than another, and these should engage our attention. To enable us to adopt the same instrument to different degrees of thickness in the calf or length in the heel, we may make use of a leather shoe, open from the toe with a wooden sole, suspended from the top of the foot-board by means of a strap and buckle. The strap should be fastened to the bottom of the wooden sole in a groove, and made to pass over the top of the foot-board, which should be furnished with a buckle near the end that comes in contact with the leg piece, in order to receive it. The strap should be let a little into the top of the foot-board, that it may not shift its situation. The sole of the shoe should be confined closely to the foot-board by a strap and buckle passed round them. The same intention may be answered by making the sole of the shoe to slide in a groove in the foot-board, upon which it may be fastened by means of a thumb-screw. The leather of the shoe is intended to wrap over the instep, and to be confined upon it with straps or ribbons.

"This shoe is intended to answer three indications:—to raise the heel from the leg-piece, or lower it, as the case may require; to prevent any lateral or rotatory motion between the fractured ends of the bones; and to form an easy bed for the heel.

"The whole of what I have described, is required for fractures of the thigh; and for fractures of the leg, attended with high inflammation; but when the instrument is used for common simple fractures of the leg, as we usually see them, the brass plate may be dispensed with.

"Besides the parts I have mentioned, three common short splints are required for fractures of the thigh: one on the outer, another on the front, and another on the inner side of the thigh. The first should extend from the upper part of the great trochanter to the lower part of the outer condyle; the second from the great trochanter to the patella; the third should lie upon the triceps adductor femoris, and should extend from the pelvis to the lower part of the inner condyle. These, with the assistance of the thigh-piece, may be made gently and regularly to compress the muscles, and to prevent any straps or tapes, made use of to secure them, from injuring the soft parts. The splint placed upon the front of the limb answers another indication, that is to resist the action of the flexors of the thigh.

"For fractures of the leg, three short splints are also required; one on the outer, another on the inner side of the leg, running from the head of the tibia or condyles of the femur to the sole of the foot. These differ but little from common short splints for fractures of the leg. Each of them has a small hole at the lower extremity, which corresponds with one on the same side of the foot-board. They are a little narrower, and the holes for the ancles are made to answer to the situation of the malleoli, the one for the inner ancle being nearer the anterior edge of the splint than that for the outer. These splints steady the foot more perfectly, and enable the patient to lay the limb upon the side. The third splint, which is composed of deal, one half of which is split, is turned up a little at either end, and is intended to lie upon the front of the leg. This prevents the upper fragment of the fracture from being at all displaced by pressure on the calf, and preserves the integuments on the shin from the action of straps or tapes.

"I have had an instrument constructed in the manner above described, and have used it in a variety of cases, and have not yet been able to discover any thing material that militates against it. In confining the different splints upon the limb, I have made use of leather straps, armed with buckles, in preference to tapes; as the necessary degree of pressure can be better and more easily regulated with the former than the latter; and as there is no danger of their giving way. In order to fix them to the instrument, or remove them at pleasure, I have usually had some studs put along the middle of the back of the instrument, and a strip of leather near each side, for the straps to pass under.

"Having described the different parts of this instrument, we now pass on to its application. This is modified by the state of the soft parts, and the situation of the fracture. The surgeon should bear in mind the principles of his science, and act up to them in every instance. If no particular angle is indicated, by the nature of the case, the semiflexed position is most easy to the patient, both in fractures of the leg, and fractures of the thigh. Whether the fracture be in the leg, or in the thigh, the limb should be placed upon the instrument, as soon as possible after the accident, and this is the more necessary, in proportion as the displacement is great, or the inflammation high; but as long as the high irritation continues, it is not adviseable to apply the short splints closely upon the fractured bone. We may indeed defer the application of the short splints to the fractured part, till the first irritation is got under, unless the bone cannot be kept in apposition from the irritability of the patient, or other causes.

"In a case of fractured leg, before the first irritation has subsided sufficiently for the application of the short splints, supposing no particular indication requires them to be immediately applied, the limb should be put up in the following manner:—The surgeon, having procured an instrument, should fit it to the sound limb. The hinge which connects the leg and thigh pieces should be placed under the knee-joint, and the foot board brought up close to the foot. He should then superintend the construction of a pad, made thickest at the part which is intended to lie under the small of the leg, and long enough to cover the leg and thigh-pieces, upon which the limb is to be placed.* A tape should be placed transversely upon the back of the foot-board near its loose end, under a strip of leather, placed there to prevent the tape from slipping down to the end of the leg-piece. The ends of this tape should then be carried from the sides of the foot-board, through the corresponding brass eyes connected to the sides of the thigh-piece, and left hanging. The instrument being now properly adjusted, two assistants should raise the fractured limb, one the foot, and the other the upper fragment, while the surgeon places the instrument, properly padded, beneath it. The instrument being in a line with the limb, and the hinge under the knee, the assistant should be directed to lower the limb gradually upon it, and place the heel in the shoe. Then, having placed a splint upon the front of the thigh, and another on the inner side upon the triceps, two or three straps should be carried round the thigh upon the splints, so as to make very little pressure upon the limb. One of the straps, drawn rather closer than the others, should pass over the condyles upon the femur, to keep the knee from rising from the instrument, when the limb is moved. The only use of the short splints, placed upon the thigh, is to prevent the straps that fix the thigh upon the instrument from injuring the soft parts. The tape, made to cross the loose end of the foot-

---

* Mr. A. now uses a leather strap instead of a tape, as it supports the foot-board more steadily, and is more easily regulated. The strap is fixed to one of the brass eyes on one side of the thigh-piece; and the buckle to the other. In applying the instrument, the strap is carried from the side of the thigh-piece, round the toe of the foot-board, under the leather placed there to support it, and then along the opposite side of the instrument, where it is received by the buckle at the side of the thigh-piece.

board, should now be drawn as tight as is comfortable to the patient, and tied upon its back. The tape is intended to keep the foot-board close against the sole of the foot, which should be left extended a little beyond a right angle with the leg. The leather of the shoe should now be laid over the instep and toes, and secured as close as the patient can bear it with ease. The limb being thus put up, the foot of the instrument should be raised, and the patient left upon his back, with the limb upon the heel. In this state, leeches, cold lotions, &c. may be applied; the patient's bowels may be freely evacuated; he may have his bed made daily, &c. without any danger of disturbing the fracture, if care is taken not to turn the instrument to either side. When the inflammation and tumefaction have subsided, the surgeon should notice the bone particularly, to see whether the fragments continue in a line, and depress or raise the shoe upon the foot-board, as circumstances may require. The tape that supports the foot-board should now be gradually drawn sufficiently close to place the foot at right angles with the leg, taking care at the same time that the bones are not made to overlap, if the fracture is oblique. The short splints, properly padded, may now be applied to the leg, with the unsplit part of the front one upon the tibia, and confined moderately close with two or three circular straps. A tape should be passed through the small hole at the lower end of each of the side splints, and then through the corresponding hole in the foot-board. These tapes should be brought together on the back of the foot-board, and tied. The use of these tapes is to prevent the lower ends of the side splints from shifting when the patient moves the limb. The straps upon the thigh having been drawn a little closer, the patient may be furnished with a sling, which should be fixed so as to act upon *the* heel of the foot-board and lower end of the leg-piece*, and desired to get up and place his limb across a chair, or walk about at pleasure on crutches, with a *caution not to move the limb but by means of the sling.* The sling should not be taken off the instrument night nor day, during the period of cure, as it gives the patient perfect command over the lower part of the limb, and enables him to place it in any position he pleases.

"In all cases of fracture of the thigh, it is necessary to make use of the apparatus for lenthening the thigh piece.

"The instrument, with the sliding plate attached to it, should be placed under the sound limb and fitted to the leg and foot, as for fractures of the leg; and the thigh-piece should be regulated so as to reach from the bend of the knee to within half an inch of the tuberosity of the ischium. The pad should be made a little longer than the instrument, that it may fall a little over its upper end, where

---

* It is of importance that this direction should be strictly attended to, for if the sling is fixed so as to move the foot-board, the motion will be communicated to the fracture, and a tardy union will be the consequence."

it should be fastened; and should be thick enough at this part, when compressed, to fill up the space left between the instrument and the tuberosity of the ischium. The surgeon should superintend the making of this pad, for it is of importance in two respects. If the thigh part be nicely adapted to the length of the thigh, the instrument will tend to keep the broken bone the same length as the sound one, by its pressure against the tuberosity of the ischium, and upper part of the back of the leg; and if its upper end be sufficiently protected, it will not inconvenience the patient by its pressure upon the soft parts.

"When there is much contusion or inflammation of the soft parts, the instrument, properly adjusted to the length of the sound limb, should be applied in the following manner:—Two assistants should raise the limb from the bed, while the surgeon places the instrument beneath it. The limb being placed upon the instrument, the foot-board should be properly fixed with the tape, and the foot secured in the shoe, as described for fractures of the leg. A couple of straps should then be passed round the leg upon the instrument, and a bit of splint padded and placed upon the shin. The leg and foot being thus secured, and a long strap with a sliding pad attached to it being previously carried between the steel bars and the brass plate, one assistant should keep the upper end of the instrument close against the back of the thigh, while the other, by extending from the knee, draws down the instrument, and with it the lower fragment, till the upper end of the instrument comes anterior to the tuberosity of the ischium, in the same manner as when it was placed upon the sound limb. The surgeon, having ascertained this point, and that the fractured ends of the bone are in apt and proper contact, the broad strap, placed at the upper end of the instrument, should now be made to cross upon the front of the thigh, pass round the pelvis, and buckle.

"This strap keeps up the instrument against the back of the thigh, and serves to secure it to the pelvis, and therefore the instrument and lower part of the limb are made to follow those motions of the pelvis which tend to disturb the fracture.

"The whole length of the thigh is left bare, except at its back part, and little or no impediment is given to the circulation. The limb thus put up should be left resting on the heel upon pillows, with the patient on his back. It is to be recollected that, if any particular indications require the limb to be secured more firmly, the short splints should be lightly applied

"As soon as the high action is sufficiently got under, which is usually in the course of two or three days, the short splints, properly padded, should be placed upon the limb and secured with three or four straps. The broad padded strap, previously passed round the thigh and the pelvis, should now be carried under the leathers, placed upon the back of the short splints, made to cross upon the outer of these splints; and again to pass round the pelvis, and buckle. This strap keeps the upper ends of all the splints firmly upon the upper fragment, and serves to connect them with the pelvis.

" If the broken ends of the bone do not ride, and if the fracture be not so high up as to prevent the upper fragment from being held firmly by the splints, the patient may now be allowed to place his limb upon the side or upon the heel, and to alter the position at pleasure; not by means of a sling, as in fractures of the leg, but by placing his finger under one of the straps surrounding the lower end of the thigh, and at the same time taking care that the pelvis and the limb move together in the same direction. The limb however had better be allowed to lie principally upon the side with the toe and knee a little raised from the bed by means of pillows, if the bone is broken just below the trochanter minor, or if the fracture is oblique.

" During the cure, it is necessary that the surgeon should attend to two points particularly. *He must see that the instrument does not ride over the tuberosity of the ischium; he must take care that the patient does not move the limb by the exertion of its own muscles*, but by the assistance of his hand, or by the assistance of another person. The reason of these two directions will immediately appear. The riding of the instrument would indicate that the fractured ends of the bone overlap; and if the patient attempts to move the limb, by its own powers, he would incur the danger of displacing the upper fragment; for the muscles surrounding the fracture are not of a texture capable of opposing much resistance to lateral displacement, which their own contraction would tend materially to produce.

" If more extension and counter-extension be required than the instrument itself is capable of effecting, which I am disposed to believe will seldom be the case, the patient should be placed on his back diagonally upon the bed. Extension may then be made by a tape, which should be fastened to the brass eyes in the sides of the thigh-piece, thrown over a pully at the foot of the bedstead, and made to suspend a weight sufficient for the purpose for which it was intended. The tape, before it passes over the pully, should take the line of the thigh part of the instrument at whatever angle it may be placed, and whether it lies upon the heel or upon the side; it will therefore be necessary for the pully to be suspended from the ceiling, the foot of the bedstead, by means of a forked stick, or any other means suited for the purpose. Counter-extension may be kept up by means of a bandage, padded and passed round the upper part of the thigh. The two ends of this bandage should be made to cross upon the ilium of the affected side; and then one end may be carried under the pillows, and the other over the chest to the opposite side of the body, where they may be fastened to the bed-post.

We now come to the cases which were treated according to the plan above related.

" I was favoured (says the author) by Mr. Travers, with the treatment of the following case of fracture of the thigh.

" *Case* 1.—Thomas Barwick, æt. twenty-six, was placed under my care December 17th, 1821, for a simple fracture of the thigh across the middle. The injury was occasioned by the wheel of a cart, which passed over the limb, considerable contusion and tension followed the accident; but the muscles were tranquil, and the fractured ends of the bone did not appear to ride.

"I saw him the fourth day from the time of the accident, and applied the machine. He had his bed made directly, and had it repeated during the cure as often as he wished; and placed his limb in any position most congenial to his feelings, either on the heel or on the sides, and altered the position at pleasure.

"January 17th, 1822, he got up with the instrument on, and walked about on crutches, supporting his limb in a sling. At the end of five weeks from the time of the accident, the machine was taken off; and, at the end of six, the man was able to walk without crutch or stick, and had a straight and perfect limb. As there was still some weakness about the muscles of the thigh, he was desired to steady himself a little longer with a crutch or stick.

"He has since informed me, that he was able to carry a sack of malt the distance of fifteen yards, ten weeks after the accident."

"This case proves, as far as any one case can prove—1st, that there is no necessity for confining the patient to one position, during the cure of a simple transverse fracture of the thigh across the middle. 2d. That allowing the patient to alter the position of the limb under the restrictions above stated, does not retard the union. 3d. That the treatment here laid down is more conducive to the comforts of the patient and to a speedy cure than any previously adopted."

"*Case* 2.—I feel indebted to Mr. Key, assistant-surgeon to Guy's Hospital, who kindly offered me the treatment of the following case:

"Mary Lovel, æt. forty-five, February 8th, 1822, slipped off the flag-stones, and, her foot twisting under her, she fell and broke the tibia and fibula just above the ancle-joint.

"Fourteen days after the accident, I saw her. The tension and pain were then very great, and the foot was lying in the extended position, where it was kept by the spasmodic action of the gastrocnemii muscles; there was an angle formed by the bones projecting forward at the seat of fracture; and the fractured end of the lower portion of the tibia threatened to come through the skin. I was informed that the symptomatic fever had run high. She had been out of bed in a delirious state. Leeches, fomentations, and cold lotions, had been applied, and had, in some measure, reduced the inflammation and tension.

"The machine was now lightly applied upon the limb. 24th. The pain and tension having materially abated, the short splints were placed upon the leg; and, the straps having been drawn moderately tight, the woman was desired to sit up with her leg across a chair. In a few days, she was able to walk about the room with the assistance of crutches. Three weeks after the application of the machine, the bones were found firmly united; and the limb was straight and perfect. The machine was now taken off, and she was desired to walk about the room with the assistance of crutches, bearing lightly upon the foot at every step. In six weeks and two days she was able to walk without crutch or stick; but did not throw aside her crutches altogether till the end of the seventh week."

" *Case* 3.—T. C. æt. fifty-five, March 28th, 1822, fell from the step of a carriage, and his foot twisting under him, the tibia was fractured obliquely into the ancle-joint, and the fibula a little above it. He was put to bed with the limb upon the side. Ten leeches were immediately applied to the part, and the bleeding promoted by fomentation.

" 29th. The machine was applied and the leg kept wet with sedative lotion. He had considerable symptomatic fever, which was removed by a strong purgative. 31st. The active inflammation having subsided, short splints were applied to the leg, and the man ordered to get up, and put his limb across a chair. April 1st, the fourth clear day of the accident; he was up yesterday, and has been walking about this morning with the assistance of crutches, supporting the limb in a sling, but not without considerable pain when the limb was hung down. The increase of pain in the fracture, occasioned by the pendant state of the limb, soon subsided, upon placing the limb in the horizontal position; and, in the course of a few days, it became immaterial to him whether the limb was placed upon the floor or upon a chair. 13th. He was seized with a pleuritic attack, which confined him to his bed three days. 22d. The twenty-fourth clear day after the accident, the instrument was taken off, and the bones were found straight and firmly united. 23d. Soap plaster and a bandage being applied, he was desired to walk about with crutches, bearing lightly upon the foot at every step. Five weeks after the accident, this man was able to walk across the room without crutch or stick, but as he had not yet full power over the muscles of the leg, he was desired to steady himself with one or the other a little longer."

" *Case* 4.—A lady of rank and fortune, received a blow from the heel of a horse, as she was on horseback, on the lower part of her stirrup leg. The force fractured the fibula about two inches and a half above its lower extremity, and the tibia, an inch and a half above the point of the inner malleolus. She applied to her surgeon in the country, who placed her in the horizontal position, and did every thing he thought advisable. Eight weeks after the accident, finding she had no power over the limb, she came to town to consult Sir Astley Cooper, and by his recommendation, requested my attendance. The fibula was, at this time, united close to the side of the tibia, and the leg deformed in consequence; but distinct crepitus and preternatural motion still remained between the fractured surfaces of the tibia. The machine was applied, and in five weeks and three days, the tibia, also, was found firmly united. During the cure, the lady walked about with the assistance of crutches, carrying the limb in a sling, received company, or took an airing in her carriage at pleasure."

" These three cases, 2d, 3d, and 4th, prove that there is no occasion to confine a patient to his bed for simple fracture of the leg, from the mere circumstance of the bones being broken.

"Case 2d shews, in particular, that the instrument made use of, is capable of keeping the upper and lower portions of a fractured leg in a proper line, notwithstanding the existence of considerable spasmodic action in the muscles, even after other means have failed.

"Case 3d goes to prove, that a patient may leave his bed a few days after the accident has occurred, provided the inflammatory action is got under, and it would appear, from the rapidity with which this man recovered, that the slight irritation kept up in the fracture, by occasionally hanging the limb down, accelerates the union of the bones."

"Case 4th shews, that the instrument is not only capable of keeping the bones in a proper line, but, also, of preventing any motion from taking place between the fractured surfaces; for it will be recollected, that this was a case where the bones were little disposed to unite; and, if motion had been produced in the seat of fracture, in consequence of her moving about, it is not likely that union would have taken place while she continued to do so. It might be said, that motion was produced between the fractured surfaces, and that this caused the bones to inflame and throw out callus; but I believe it will be found, as I shall hereafter endeavour to shew, that motion alone is little calculated to produce union in bone, any more than between the divided surfaces of any other texture."

The author states, that it is his intention to give an account of the use of his instrument in the treatment of compound fractures and cases of non-union, and, also, in the treatment of injuries of the knee and dislocations of the ancle; but he reserves the consideration of these subjects till some future period. In the mean time, as we have heard the apparatus well spoken of by some good judges, we have presented the public a full account of it at our own private expense.

